# Luteolin Reduces Alzheimer’s Disease Pathologies Induced by Traumatic Brain Injury

**DOI:** 10.3390/ijms15010895

**Published:** 2014-01-09

**Authors:** Darrell Sawmiller, Song Li, Md Shahaduzzaman, Adam J. Smith, Demian Obregon, Brian Giunta, Cesar V. Borlongan, Paul R. Sanberg, Jun Tan

**Affiliations:** 1 James A. Haley Veteran’s Administration Hospital, Tampa, FL 33612, USA; 2 Department of Psychiatry, Morsani College of Medicine, University of South Florida, Tampa, FL 33613, USA; E-Mails: songli@mail.usf.edu (S.L.); dobregon@health.usf.edu (D.O.); bgiunta@health.usf.edu (B.G.); jtan@health.usf.edu (J.T.); 3 Department of Biophysics, School of Physics and Optoelectronic Technology, Dalian University of Technology, Dalian 116024, China; 4 Center of Excellence for Aging and Brain Repair, Department of Neurosurgery and Brain Repair, Morsani College of Medicine, University of South Florida, Tampa, FL 33613, USA; E-Mails: mshahad@health.usf.edu (M.S.); asmith1@health.usf.edu (A.J.S.); cborlong@health.usf.edu (C.V.B.); psanberg@health.usf.edu (P.R.S.)

**Keywords:** traumatic brain injury, Alzheimer’s disease, amyloidogenesis, tauopathy, GSK, neuroinflammation, luteolin

## Abstract

Traumatic brain injury (TBI) occurs in response to an acute insult to the head and is recognized as a major risk factor for Alzheimer’s disease (AD). Indeed, recent studies have suggested a pathological overlap between TBI and AD, with both conditions exhibiting amyloid-beta (Aβ) deposits, tauopathy, and neuroinflammation. Additional studies involving animal models of AD indicate that some AD-related genotypic determinants may be critical factors enhancing temporal and phenotypic symptoms of TBI. Thus in the present study, we examined sub-acute effects of moderate TBI delivered by a gas-driven shock tube device in Aβ depositing Tg2576 mice. Three days later, significant increases in b-amyloid deposition, glycogen synthase-3 (GSK-3) activation, phospho-tau, and pro-inflammatory cytokines were observed. Importantly, peripheral treatment with the naturally occurring flavonoid, luteolin, significantly abolished these accelerated pathologies. This study lays the groundwork for a safe and natural compound that could prevent or treat TBI with minimal or no deleterious side effects in combat personnel and others at risk or who have experienced TBI.

## 1. Introduction

Traumatic brain injury (TBI) occurs in response to an acute insult to the head, such as after firearm injury, motor vehicle accident or fall, and is recognized as a major risk factor for Alzheimer’s disease [1–3]. Indeed, recent studies have suggested a pathological overlap between TBI and AD. For example, long-term survivors of just a single moderate to severe TBI (1–47 years survival; *n* = 39) exhibited abundant and widely distributed neurofibrillary tangles (NFTs) and Aβ plaques in approximately one-third of the cases but this was exceptionally rare in uninjured controls [4]. In addition, the plaques found in TBI patients are strikingly similar to those observed in the early stages of AD [5,6]. Plaques in AD develop slowly and are typically found in the elderly, whereas TBI-associated plaques can be seen within hours post-TBI. The major form of Aβ in plaques and of soluble Aβ generated after TBI and AD is Aβ42, which is prone to aggregation and neurotoxicity [7,8]. An additional post mortem study found that brains from military veterans with blast exposure and/or concussive injury exhibited similar neuropathology to that found in young adult athletes with histories of repeated concussive injuries, including tau neuropathology, neuroinflammation and neurodegeneration [9].

Animal models provide further evidence for a link between TBI and AD. Within two weeks after exposure to a single controlled blast, wild-type C57BL/6 mice demonstrated phosphorylated tauopathy, chronic neuroinflammation, neurodegeneration, and persistent hippocampal-dependent learning and memory deficits as seen in AD [9]. In PSAPP (SweAPP, PSEN1dE9) AD mice, which carry human Swedish mutant amyloid precursor protein (SweAPP) and mutant presenilin 1 (PS1) genes, a single controlled cortical impact (CCI) injury precipitated cognitive impairment and extracellular Aβ deposits between two and six weeks post- injury [10]. Studies of triple-transgenic mice expressing mutations in human tau and PS1, as well as SweAPP, have shown that a single CCI injury can lead to a rapid accumulation of hyperphosphorylated tau and intra-axonal Aβ within 24 h after experimental injury [11,12]. These findings suggest that some AD related genotypic determinants may be critical factors enhancing temporal and phenotypic symptoms of TBI. They also highlight the need for the development of novel therapies to abrogate cellular injury, tauopathy, and Aβ deposits in the treatment of TBI.

Recent focus has been given to a group of polyphenols categorized as flavonoids, which have been found by our group and others to be potentially antiamyloidogenic [13]. We previously found treatment of murine N2a cells transfected with SweAPP (SweAPP N2a cells) and primary neuronal cells derived from SweAPP overexpressing mice (Tg2576 line) with the flavonoid luteolin results in a significant reduction in Aβ generation through selective inactivation of the GSK-3α isoform [13]. This inactivation of GSK-3α increases phosphorylation of PS1, which forms the catalytic core of the γ-secretase complex, thereby reducing PS1-APP interaction and Aβ generation. These results suggest a mechanism whereby these small-molecular weight compounds (GSK-3 inhibitors) reduce AD pathology. Importantly, this naturally occurring flavonoid does not inhibit Notch processing as assessed by Western blot analysis. Furthermore, luteolin has been shown to attenuate zinc-induced tau hyperphosphorylation in SH-SY5Y cells through not only its antioxidant action but also by inhibition of the tau kinase p7056K and recovery of total phosphatase activity [14]. Since TBI can manifest AD-like pathological features, we examined if luteolin could abolish these features using the amyloid depositing Tg2576 mouse model of AD.

## 2. Results and Discussion

### 2.1. Luteolin Significantly Reduces Amyloid Pathology Elicited by TBI in Tg2576 Mice (Figure 1a–c)

**Figure 1.**
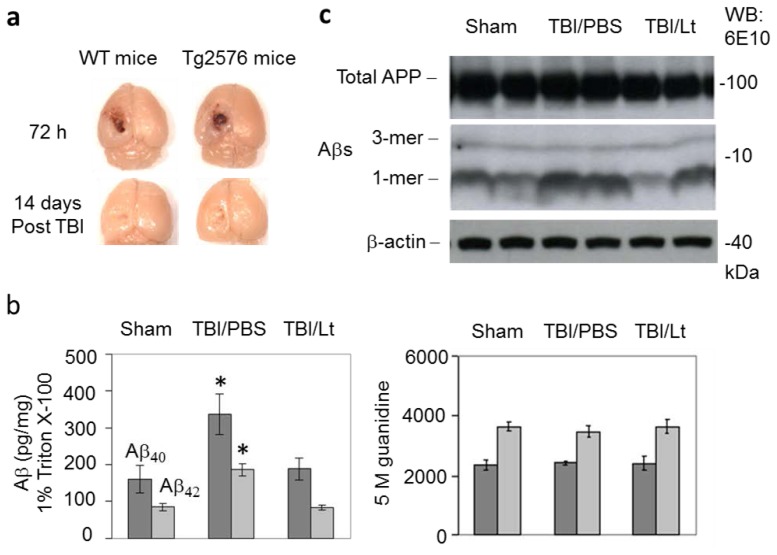
Luteolin significantly reduces amyloid pathology elicited by traumatic brain injury (TBI) in Tg2576 mice. TBI was elicited in Tg2576 mice and wild-type (WT) littermates using a controlled cortical impactor. A single impact trauma was delivered with a 1.0 mm impact probe, an impact depth of 1.0 mm, velocity of 6 m/s and dwell time of 100 ms. Following 72 h and 14 days post injury, the mice were sacrificed and the damaged area was determined by thionin coloration. The area of brain damage at 72 h and 14 days following TBI were similar between Tg2576 and WT controls (**a**); For determination of amyloid pathology, Tg2576 mice (*n* = 12, 6 F/6 M) were treated with luteolin (Lt) at 20 mg/kg or PBS ip daily for 15 days followed by TBI. Three days after TBI, the mice were sacrificed and brain homogenates were prepared and subjected to (**b**) ELISA for cerebral Aβ levels and (**c**) western blot (WB) for analysis of total APP and Aβ monomers and trimers using an anti-Aβ_1–17_ antibody (6E10). As shown, TBI significantly increased soluble Aβ_40,42_ levels, extracted with 1% triton X-100 (asterisks indicate *p* < 0.01), and this increase was solely due to an increase in Aβ monomers. In addition, luteolin abolished this increase in soluble Aβ levels. TBI did not increase insoluble Aβ_40,42_ levels, extracted with 5 M guanidine.

The area of brain damage at 72 h and 14 days following moderate TBI were similar between Tg2576 and WT controls (Figure 1a). TBI significantly increased soluble Aβ_40,42_ levels in PBS pretreated mice (*p* < 0.01), extracted with 1% triton X-100, and this effect was significantly blunted by luteolin pretreatment (*p* < 0.01) (Figure 1b). Western blot analysis indicated that this increase in Aβ levels was solely due to an increase in monomeric Aβ (Figure 1c). TBI did not significantly increase insoluble Aβ_40,42_, extracted with 5 M guanidine. Therefore, short term TBI accelerates Aβ pathology in the Tg2576 mouse model of AD and luteolin pretreatment ameliorates this effect.

### 2.2. Luteolin Significantly Reduces GSK Activation, Tau Phosphorylation and Microglial-Induced Release of Inflammatory Cytokines Elicited by TBI in Tg2576 Mice (Figure 2a,b)

TBI but not sham treatment significantly increased AD-like active phosphorylated GSKβ, and phospho-tau, as determined by western blot, and levels of microglial-derived inflammatory cytokines, TNFα and IL-1β, as determined by ELISA (*p* < 0.01). Moreover, these effects were significantly abolished with luteolin pretreatment (*p* < 0.01).

### 2.3. Luteolin Is Brain Permeable (Figure 2c)

Finally, we determined the ability of peripherally administered luteolin to cross the blood brain barrier (BBB). Non-transgenic littermates were treated with luteolin at 1.25, 2.5, 5 and 10 mg/kg/day by gavage for 4 weeks followed by preparation of blood and brain tissues for total luteolin analysis by HPLC [15,16]. Total luteolin levels in blood and brain tissues reached approximately 800 ng/mL after 4 weeks treatment at 5 and 10 mg/kg/day. Altogether, these results indicate that peripheral administration of luteolin freely penetrates the BBB and enters the brain, underscoring the potential effectiveness of luteolin for treatment of TBI-induced AD pathology.

These studies indicate that luteolin pretreatment administered peripherally can reduce TBI-elicited AD pathology, as shown by reduced Aβ deposition, tau hyperphosphorylation, GSK activation and microglial proinflammatory cytokines. Previous studies have shown that axonal injury is one of the most common pathologies of TBI and independently contributes to significant morbidity and mortality [17–19]. It entails interruption of axonal transport due to cytoskeletal disruption, which can also cause axonal accumulation of APP and subsequent formation of Aβ. Eventual structural axonal disruption can lead to secondary axonal disconnection, ending in degenerative axonal pathology. Indeed due to rapid build-up of APP in damaged axons after TBI, APP immunostaining is used for the pathological assessment of diffuse axonal injury in humans. Accordingly, it was suspected that this large reservoir of APP in injured axons might be amyloidogenically cleaved to rapidly form Aβ [20]. In addition, immunohistochemical analyses showed that γ- and β-secretases necessary for amyloidogenic Aβ cleavage also accumulate in injured axons after TBI [15]. Notably, at a much slower rate, this general process of axonal transport breakdown has been implicated as a mechanism of amyloidogenic APP processing in AD [16]. It has also been postulated that elevated APP production in the neuronal soma after TBI may saturate the normal α-secretase processing pathway, resulting in increased amyloidogenic β- and γ-secretase processing and Aβ genesis [8,21].

Like Aβ, neurofibrillary tangles (NFT) have also been implicated as a central pathological feature of AD. They are composed of misfolded and hyperphosphorylated tau, a microtubule protein [22,23]. In the Tg2576 mouse model of AD, NFT-like, abnormal hyperphosphorylated tau have been shown to accumulate [24,25]. In addition, the accumulation of Aβ due to TBI can adversely affect distinct molecular pathways, facilitating tau phosphorylation, aggregation, and NFT accumulation [26]. Aβ and abnormal hyperphosphorylated tau can also synergize to accelerate neurodegenerative mechanisms, impairing metabolism, cellular detoxification and mitochondrial function, ultimately resulting in neuritic plaque formation [27].

A pathological link between TBI and AD is further established by a condition known as chronic traumatic encephalopathy (CTE), also known as dementia pugilistica [28–30]. This neurodegenerative condition has been described in the brains of boxers and more recently found to be caused by other forms of repetitive concussive head injury, such as that which occurs in response to war battle, American football, hockey, soccer or physical abuse. These changes are typified by (1) cerebral atrophy; (2) cavum septum pellucidum with fenestrations; (3) shrinkage of the mammillary bodies; (4) dense tau immunoreactive inclusions (neurofibrillary tangles [NFT], glial tangles and neuropil neurites); (5) diffuse axonal injury; and, (6) late in the disease process, a deposition of brain amyloid beta (Aβ) plaques as seen in Alzheimer’s disease (AD). In association with these pathological changes, affected individuals often exhibit disordered memory and executive functioning, behavioral and personality disturbances, parkinsonism and, occasionally, motor neuron disease. At the present time, there are no formal clinical or pathological diagnostic criteria for CTE, but the distinctive neuropathological profile of the disorder lends promise for future research into its prevention, diagnosis, and treatment [31]. The mechanisms of injury and biological basis underpinning TBI and sequelae, leading to CTE or even full-blown Alzheimer’s disease (AD), are a matter of significant controversy. As such, uncovering these mechanisms to pinpoint treatment targets is a major priority for this field.

## 3. Experimental Section

### 3.1. TBI Procedure

In our preliminary studies, we established the surgical procedures to induce three levels of TBI in Tg2576 mice and littermate controls (WT) using a compressed gas controlled cortical impactor (Leica Microsystems, Buffalo Grove, IL, USA, #39463920). The mice were deeply anesthetized with isoflurane and then mounted onto a stereotaxic apparatus to ensure an even position. A 1 cm skin incision was made from lambda to just rostral to bregma and the soft tissue was removed from the surface of the skull. A 3 mm diameter window was made over the left frontoparietal cortex using an electric rotary drill at −2.0 mm anteroposterior and +2.0 mm mediolateral to bregma. The impactor with a tip diameter of 0.5 mm (mild TBI), 1.0 mm (moderate TBI) or 1.5 mm (severe TBI) was positioned at an angle 15° to the vertical to maintain a perpendicular position and the zero depth position was determined by slowly lowering the impact tip until it just touched the surface of the exposed dura mater. A single impact trauma was delivered with an impact depth of 1.0 mm, velocity of 6 m/s and dwell time of 100 ms. Mice were then carefully removed from the stereotaxic frame, the incision was stapled and the mice were allowed to recover from anesthesia on a heated pad. The mice were then returned to their home cages. Following 72 h and 14 days post injury, the mice were sacrificed and the damaged area was determined by thionin coloration. We found that the 1.0 mm probe tip, producing moderate TBI, produced profoundly enhanced cerebral Aβ levels and therefore the remaining mice in this study were subjected to this level of TBI.

### 3.2. Luteolin Administration

For determination of the effect of luteolin on TBI acclerated AD pathology, Tg2576 mice were treated with luteolin at 20 mg/kg ip or PBS daily for 15 days based on previously described methods [13] followed by induction of TBI. In total, 12 mice (6 F/6 M) were used for this determination, 6 received luteolin and 6 received PBS. An additional 6 Tg2576 mice were subjected to sham TBI as control, which were treated like those subjected to TBI but the impactor driving the probe tip was not activated.

### 3.3. ELISA

Three days after TBI or sham TBI, all mice were sacrificed. Homogenates were prepared from the left half of brain tissues and subjected to 1% triton X-100 or 5 M guanidine extraction for determination of soluble and insoluble Aβ40, 42 by ELISA, respectively. ELISA was also used for determination of various microglial proinflammatory cytokines, including TNF-α and IL-1β.

### 3.4. Western Blot Analysis

Western blot was performed as described previously [10,13,32]. Briefly, brain homogenates were lysed in ice-cold lysis buffer and aliquots were electrophoretically separated using 16.5% Tris-tricine gels. Electrophoresed proteins were then transferred to PVDF membranes (Bio-Rad, Hercules, CA, USA), washed in dH_2_O, then blocked in Tris-buffered saline containing 5% (*w*/*v*) non-fat dry milk. Membranes were then hybridized with various primary antibodies followed by washing in dH_2_O and incubation for 1 h at ambient temperature with the appropriate HRP-conjugated secondary antibody (1:1000). Blots were developed and analyzed using the Fluor-S MultiImager™ and Quantity One software (Bio-Rad). Primary antibodies used included AT270 (total and phosphorylated tau, Thermo Scientific, Rockford, IL, USA), 6E10 monoclonal anti-Aβ antibody (total APP and Aβ, Covance, Emeryville, CA, USA), pTyr279/216 (total and phosphorylated GSK, Sigma Aldrich, St. Louis, MO, USA) and anti-actin antibody (Sigma Aldrich).

### 3.5. Brain Biodistribution Study

In order to determine if peripherally administered luteolin could penetrate the blood brain barrier and enter the brain, we orally treated non-transgenic littermates with luteolin at 1.25, 2.5, 5 and 10 mg/kg/day for 4 weeks utilizing a gavage route. At sacrifice, blood and brain tissues were collected and prepared for total luteolin analysis by HPLC, as described previously [32,33]. In total, 6 mice (3 F/3 M) were used for this analysis.

### 3.6. Statistical Analysis

All data were normally distributed, followed by analysis of variance (ANOVA) and *post-hoc* comparisons using Bonferonni’s correction. Alpha levels were set at 0.05 for all analyses. The statistical package for the social sciences release 10.0.5 (SPSS Inc., Chicago, IL, USA) was used for all data analysis.

## 4. Conclusions

In summary, the present study demonstrated for the first time that luteolin can abolish AD-like features after TBI in an amyloid-β depositing mouse model. This study lays the groundwork for a safe and natural compound that could prevent or treat TBI with minimal to no deleterious side effects in combat personnel and others at risk of, or who have experienced, TBI. The Tg2576 mice subjected to TBI at various ages in both prophylactic and therapeutic paradigms would be useful in beginning to explore this possibility. Further studies should determine the effectiveness of luteolin in reducing TBI-elicited behavioral deficits. A true mechanistic understanding of what increases the risk of developing AD after TBI will also be extremely important for the development of post-trauma interventions and prophylactics aimed at halting or dampening the onset of such debilitating neurodegeneration.

## Figures and Tables

**Figure 2.**
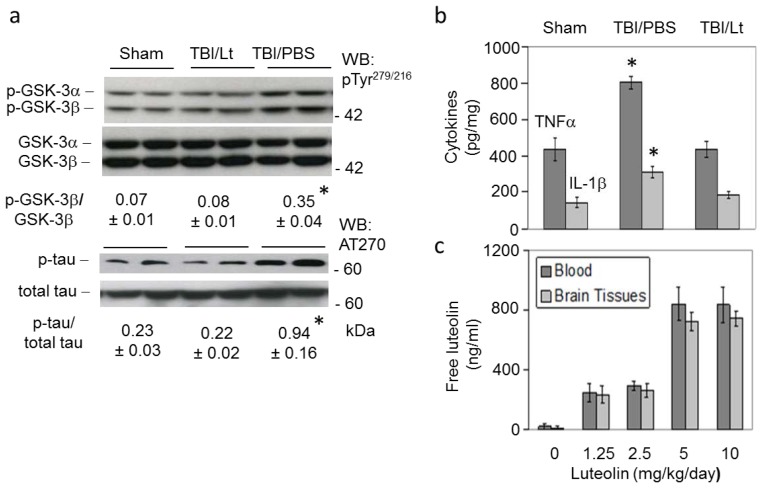
Luteolin significantly reduces GSK activation, tau phosphorylation and microglial-induced release of inflammatory cytokines elicited by TBI in Tg2576 mice. (**a**) Brain homogenates were subjected to western blot (WB) for GSK activation and tau phosphorylation using antibodies against phospho- and total GSK-3α/β (pTyr279/216) and phospho- and total tau (AT270), respectively. Densitometry analysis reveals the ratio of active phosphorylated GSK-3β to total GSK-3β and AD-like phospho-tau to total tau, as indicated below the figures; In addition, brain homogenates were subjected to (**b**) ELISA for various cytokines, including microglial-derived TNFα and IL-1β. TBI significantly increased activated phospho- to total GSK-3β ratio, phospho- to total tau ratio and TNF-α and IL-1β levels (Asterisk indicates *p* < 0.01) and these increases were significantly abolished with luteolin; (**c**) Additional studies determined the ability of peripherally administered luteolin to cross the blood brain barrier (BBB). Total luteolin levels in blood and brain tissues reached approximately 800 ng/mL after treatment with luteolin at 5 and 10 mg/kg/day by gavage for 4 weeks. Altogether, these results indicate that peripheral administration of luteolin freely penetrates the BBB and enters the brain, underscoring the potential effectiveness of luteolin for treatment of TBI-induced AD pathology.
